# Blocking K-Ras Interaction With the Plasma Membrane Is a Tractable Therapeutic Approach to Inhibit Oncogenic K-Ras Activity

**DOI:** 10.3389/fmolb.2021.673096

**Published:** 2021-06-16

**Authors:** Karen M. Henkels, Kristen M. Rehl, Kwang-jin Cho

**Affiliations:** Department of Biochemistry and Molecular Biology, School of Boonshoft School of Medicine, Wright State University, Dayton, OH, United States

**Keywords:** K-Ras, plasma membrane, mislocalization, cancer, recycing endosome, phosphatidylinositol, phosphatidylserine, sphingomyelin

## Abstract

Ras proteins are membrane-bound small GTPases that promote cell proliferation, differentiation, and apoptosis. Consistent with this key regulatory role, activating mutations of Ras are present in ∼19% of new cancer cases in the United States per year. K-Ras is one of the three ubiquitously expressed isoforms in mammalian cells, and oncogenic mutations in this isoform account for ∼75% of Ras-driven cancers. Therefore, pharmacological agents that block oncogenic K-Ras activity would have great clinical utility. Most efforts to block oncogenic Ras activity have focused on Ras downstream effectors, but these inhibitors only show limited clinical benefits in Ras-driven cancers due to the highly divergent signals arising from Ras activation. Currently, four major approaches are being extensively studied to target K-Ras–driven cancers. One strategy is to block K-Ras binding to the plasma membrane (PM) since K-Ras requires the PM binding for its signal transduction. Here, we summarize recently identified molecular mechanisms that regulate K-Ras–PM interaction. Perturbing these mechanisms using pharmacological agents blocks K-Ras–PM binding and inhibits K-Ras signaling and growth of K-Ras–driven cancer cells. Together, these studies propose that blocking K-Ras–PM binding is a tractable strategy for developing anti–K-Ras therapies.

## Introduction

*RAS* genes were initially identified as the viral oncogenes of acute transforming retroviruses, and it was designated as a mammalian proto-oncogene when mutated *RAS* genes were discovered in human cancer cells (Barbacid, 1987). There are three main Ras isoforms—H-, N-, and K-Ras—in mammalian cells, and each is encoded by a different gene. *H-*, *N-*, and *K-RAS* are situated on chromosomes 11 (11p15.1-p15.5), 1 (1p22-p32), and 12 (12p12.1-pter), respectively (Barbacid, 1987). There are four exons that code for *H-* and *N-RAS*, while in *K-RAS*, there are two alternative fourth exons, exons 4A and 4B, that yield two splice variants, *K-Ras4A* and *K-Ras4B* (Barbacid, 1987). While H-, N-, and K-Ras4B are ubiquitously expressed in mammalian cells, K-Ras4A is precisely and spatiotemporally expressed in the murine lung, liver, and kidney (Pells et al., 1997). Knockout studies showed that neither *H-* nor *N-RAS* individually or in concert are required for normal murine embryogenesis (Esteban et al., 2001), whereas *K-RAS* is unequivocally crucial to embryonic development (Johnson et al., 1997; Koera et al., 1997). Intriguingly, K-Ras knockout mice with spatiotemporally controlled expression of H-Ras by the *K-Ras* promoter have their embryonic lethality restored but develop dilated cardiomyopathy associated with arterial hypertension at an older age, reflecting the different molecular functions of Ras isoforms in the cell (Potenza et al., 2005).

While the three Ras isoforms are nearly identical, sharing ∼90–100% homology in their N-terminal catalytic domain sequences, there is a considerable lack of homology in the C-terminal hypervariable region (HVR) of each isoform, which accounts for <15% homology being shared between any two isoforms (Hancock, 2003). These HVRs consist of two different signal sequences that allow Ras proteins to traffic to and interact with the inner leaflet of the plasma membrane (PM) (Hancock et al., 1989). The CAAX motif, the first signal sequence, is constituted by the last four amino acid residues in the HVR and is shared in common between the different Ras isoforms. For CAAX, C is cysteine, A is an aliphatic amino acid, and X is either serine or methionine (Hancock et al., 1989). Newly synthesized Ras GTPases are cytosolic and require a series of posttranslational modifications of the CAAX motif for interacting with endomembranes. First, the CAAX motif is farnesylated by a cytosolic farnesyltransferase (FTase) that covalently attaches a farnesyl group to the cysteine residue *via* a thioether bond. Farnesylated Ras interacts with the cytosolic leaflet of the endoplasmic reticulum (ER), where the AAX tripeptide is removed by the Ras and a-factor–converting enzyme (Rce1). The now C-terminal cysteine is methylated by isoprenylcysteine carboxyl methyltransferase (Icmt) (Hancock et al., 1989). The CAAX motif must be processed in this series of steps in order to maintain the correct forward trafficking of Ras isoforms, since knockout of either Rce1 or Icmt results in Ras mislocalization to the cytosol (Kim et al., 1999; Lau et al., 2014).

While the correctly modified CAAX motif can direct Ras to the ER and other endomembranes, the presence of the second C-terminal signal motif is required for maximal membrane affinity and PM localization (Hancock et al., 1990). The second signal sequence situated within the HVR varies between the different Ras isoforms such that both H-Ras, N-Ras, and K-Ras4A are palmitoylated (Cys181 and Cys184 for H-RasCys181 for N-Ras and Cys180 for K-Ras4A), while K-Ras4B has a stretch of six lysine residues, forming a polybasic domain (PBD) (Lys175-180) (Hancock et al., 1990). Palmitoylation of H- and N-Ras by the Ras palmitoyltransferase takes place in the ER and Golgi complex, where H- and N-Ras are transported *via* the classical secretory pathway to the PM (Apolloni et al., 2000). While palmitoylation of H- or N-Ras is a short-lived modification with rapid kinetics (t_1/2_ of <20 min), the depalmitoylation/repalmitoylation machinery is important for delivering consistent H- and N-Ras distribution between the Golgi and the PM at a steady state (Rocks et al., 2005; Rocks et al., 2010). Palmitoylated Ras proteins diffuse from the PM to other endomembrane compartments to reach equilibrium, but depalmitoylation by poorly characterized thioesterases enhances the rate of diffusion, and thereby promotes their continuous redirection to the ER and Golgi for repalmitoylation and unidirectional trafficking back to the PM (Rocks et al., 2005; Rocks et al., 2010). The exact mechanism on how posttranslationally modified K-Ras4B (hereafter K-Ras) is transported from the ER to the PM is not fully characterized. Recent studies have demonstrated that the delta subunit of cGMP phosphodiesterase 6 (PDE6δ) functions, in part, as a K-Ras chaperone to maintain K-Ras–PM localization. PDE6δ binds the farnesyl moiety of cytosolic K-Ras, which is released in perinuclear membranes by the release factors Arl2 and 3, from where it is trapped on the recycling endosome (RE) by electrostatic interaction, and it returns to the PM *via* vesicular transport (Ismail et al., 2011; Chandra et al., 2012; Schmick et al., 2014). Once K-Ras is transported to the PM, it binds the PM through an electrostatic interaction of the strong positive charge of the C-terminal PBD with anionic phospholipid head groups in the inner PM leaflet (Yeung et al., 2008; Zhou et al., 2017).

## K-Ras and Cancer

Oncogenic mutations in Ras are found in about 18.7% of new cancer cases in the United States per year (1.3% for H-Ras, 3.1% for N-Ras, and 14.3% for K-Ras) (Prior et al., 2020). While the oncogenic mutant K-Ras is found in approximately 88% of pancreatic, 50% of colorectal, and 32% of lung cancers (Prior et al., 2020), no anti–K-Ras drugs are currently available in clinics. Human cancer cells harboring oncogenic mutant K-Ras reprogram their signaling network so that their survival and growth depend on oncogenic K-Ras signaling, a phenomenon called K-Ras addiction (Weinstein and Joe, 2008; Singh et al., 2009; Hayes et al., 2016). RNAi-mediated knockdown of oncogenic mutant K-Ras blocks cell survival and growth in a range of pancreatic and non-small-cell lung cancers (NSCLC), which provides the rationale that blocking oncogenic K-Ras activity is a valid approach to treat K-Ras-dependent cancers. Recently, two new K-Ras direct inhibitors have shown promising outcomes in clinical trials. AMG 510 and MRTX849 are small molecules that bind to the GDP-bound inactive K-RasG12C mutant and form a covalent bond to the mutant Cys, which locks K-Ras in the inactive conformation, resulting in blocked oncogenic signaling (Ostrem et al., 2013). These compounds exhibited pronounced anticancer effects in K-RasG12C tumor mice models and clinical trials with lung and colorectal cancer patients harboring the K-RasG12C mutant (Canon et al., 2019; Hallin et al., 2020). Despite the promising clinical outcome of these inhibitors, they are specific to the K-RasG12C mutant, which is found in ∼3% of pancreatic, ∼4% of colorectal, and ∼13% of lung cancers that harbor any oncogenic mutations in K-Ras (Cox et al., 2014; Prior et al., 2020), suggesting that these inhibitors would be suitable only for a small portion of cancer patients with the oncogenic mutant K-Ras.

In addition to K-RasG12C–specific direct inhibitors, there are three other approaches that are currently being investigated for blocking all oncogenic mutant K-Ras activity. They are 1) blocking K-Ras interaction with the PM, 2) inhibiting K-Ras downstream effectors, and 3) dysregulating cell energy metabolism. This review will focus on mechanisms that regulate the PM localization of K-Ras, which could be tractable targets for developing new anti–K-Ras therapeutics.

## Dissociating Ras From the Plasma Membrane Blocks its Signal Transduction

### Preventing Ras Prenylation Dissociates Ras From the PM and Inhibits Ras Signaling

Point mutations in the CAAX motif, which block posttranslational modification, prevent Ras–PM localization and completely inhibit all biological activities of oncogenic mutant Ras (Willumsen et al., 1984). Thus, farnesyltransferase inhibitors (FTIs) were designed to phenotypically mimic this mode of Ras inhibition. FTIs demonstrated marked antitumor activity in H-Ras–driven *in vivo* and *in vitro* models, which allowed phase I studies on FTIs in 1999, with some progressing to phase III clinical trials in 2002 (Baines et al., 2011). However, FTIs were ineffective with regard to pancreatic cancers in phase II and III clinical trials in which oncogenic mutant K-Ras was found in 88% of all pancreatic cancers (Cohen et al., 2003; Van Cutsem et al., 2004; Macdonald et al., 2005). It is because in FTI-treated cells, an alternative prenyltransferase, geranylgeranyltransferase (GGTase), efficiently attaches the more hydrophobic C20 geranylgeranyl moiety to K- and N-Ras, allowing K- and N-Ras to interact with the PM and conduct a signal transduction that is equipotent with the farnesylated forms (Baines et al., 2011). Concomitant inhibition of FTase with GGTase to completely block prenylation of K- and N-Ras has been tested, but this approach has suffered from dose-limiting toxicities (O'Bryan, 2019). Also, there are more than 100 proteins that are prenylated, and these combined inhibitors would induce prohibitive off-target effects, preventing their clinical effectiveness. A recent study has demonstrated a promising strategy to specifically inhibit K-Ras prenylation. A modified FTI with an electrophilic moiety specifically interacts with the CAAX motif of K-Ras but not H-Ras, resulting in the blockage of K-Ras farnesylation and geranylgeranylation, trapping K-Ras in the cytosol (Novotny et al., 2017). Further improvements of this approach could lead to a more potent inhibitor of K-Ras prenylation and activity (Novotny et al., 2017; O'Bryan, 2019).

### Perturbing K-Ras/PDE6δ Interaction Blocks K-Ras–PM Binding and K-Ras Signaling

**FIGURE 1:**
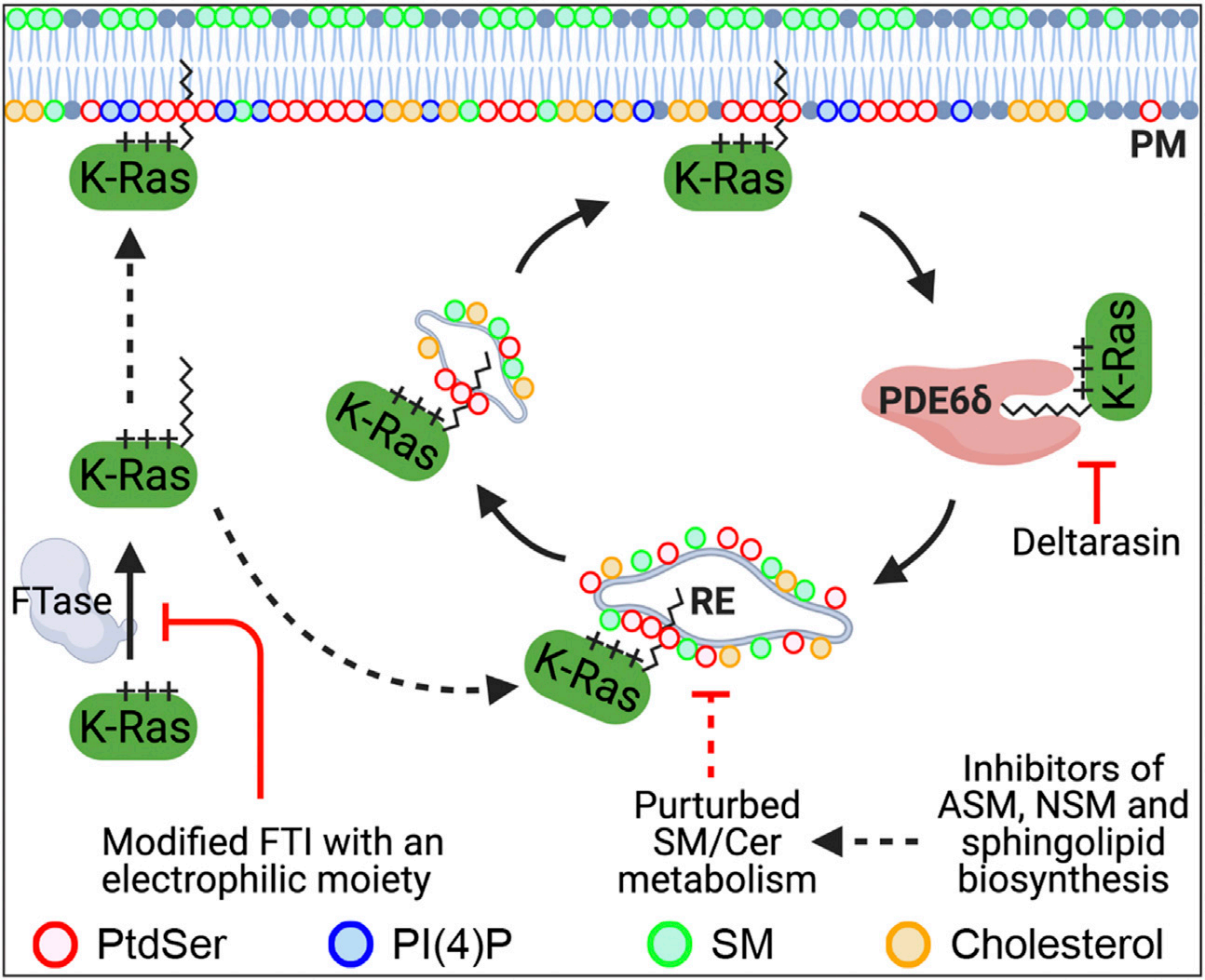
Recently identified molecular mechanisms that regulate the PM localization of K-Ras. K-Ras farnesylated by FTase localizes to the PM. Once K-Ras dissociates from the PM, PDE6δ binds K-Ras *via* its farnesyl moiety and releases it in the perinuclear region. K-Ras is then translocated to the recycling endosome (RE) through electrostatic interaction, where it returns to the PM *via* RE-mediated vesicular transport. Blocking K-Ras prenylation or the K-Ras/PDE6δ interaction mislocalizes K-Ras from the PM. Perturbed SM/ceramide metabolism is proposed to dysregulate the RE *via* altering its lipid composition, resulting in depletion of PtdSer and K-Ras from the PM. FTase, farnesyltransferase; FTI, FTase inhibitor; PDE6δ, phosphodiesterase 6 δ; RE, recycling endosome; PtdSer, phosphatidylserine; SM, sphingomyelin; Cer, ceramide; PI4P, phosphatidylinositol 4-phosphate; ASM, acid sphingomyelinase; NSM, neural sphingomyelinase.

Recent studies have shown that blocking PDE6δ interaction with K-Ras is a tractable strategy to inhibit K-Ras–PM localization and oncogenic K-Ras signaling. PDE6δ binds the farnesyl moiety of K-Ras *via* its hydrophobic pocket and acts in part as a chaperone. The release factors Arl2 and 3 unload K-Ras from PDE6δ in the perinuclear region, whence K-Ras binds to the recycling endosome (RE) for redelivery to the PM *via* vesicular transport (Chandra et al., 2012; Schmick et al., 2014). Deltarasin is a small molecule that binds to the hydrophobic pocket and inhibits PDE6δ/K-Ras interaction, resulting in K-Ras–PM mislocalization and abrogated signaling in K-Ras–driven cancer cells (Figure 1 and Table 1) (Zimmermann et al., 2013). Second-generation PDE6δ inhibitors, which bind PDE6δ more tightly *via* extra hydrogen bonds, have demonstrated greater potency for blocking the growth of K-Ras–dependent but not K-Ras–independent pancreatic cancer cells (Papke et al., 2016; Martin-Gago et al., 2017). Moreover, deltarasin does not inhibit the growth of cells transformed with the oncogenic mutant B-Raf or the overexpressed epidermal growth factor receptor (EGFR) (Klein et al., 2019), suggesting that PDE6δ inhibitors are effective against K-Ras–dependent cancer cells. In addition, deltarasin functions independent of K-Ras, where it promotes autophagy by activating the AMPK/mTOR pathway, and concomitant inhibition of autophagy and PDE6δ potentiates deltarasin-mediated cell death by elevating reactive oxygen species (ROS) (Leung et al., 2018). These observations suggest that deltarasin elevates cellular ROS, which promotes autophagy (Zhang et al., 2016), and that deltarasin in combination with an autophagy inhibitor can be a plausible strategy for treating K-Ras–driven cancers (Leung et al., 2018).

**TABLE 1:** Summary of the compounds that inhibit K-Ras interaction with the PM.

**Drug**	**Target mechanism**	**Cell lines tested**	**References**
Deltarasin	Blocking interaction of PDE6 delta with farnesylated small GTPases	Panc-Tu-1, Capan-1, MIA-PaCa2, SW480, HCT-116, Hke3, A549, and H358	Zimmermann et al. (2013), Papke et al. (2016), Martin-Gago et al. (2017), Leung et al. (2018), Klein et al. (2019), O'Bryan (2019)
Staurosporine and its analogs	Perturbing endosomal recycling of PtdSer and depleting PtdSer PM content	MDCK and CHO	Cho et al. (2012b), Maekawa et al. (2016)
Fendiline and antidepressants	Functional inhibitor of ASM and depleting PtdSer PM content	MIA-PaCa2, MOH, HPAC, MPanc96, Hec-1a, Hec-1b, Hec50, NCI H23, SK-CO-1, SW948, SW1116, and Ca-Co2	van der Hoeven et al. (2013), Cho et al. (2016)
Avicin and its analogs	Inhibiting NSM and ASM	Jurkat, U2OS, NB4, AsPC-1, Panc10.05, MIA-PaCa2, HPAFII, Panc-1, H358, and H441	Wang et al. (2010), Garrido et al. (2020)
AMG510	Forms covalent bond with Cys in the K-RasG12C mutant, locking it in its inactive, GDP-bound form	H1792, H358, H23, Calu-1, MIA-PaCa2, NCI-H1373, NCIH 2030, NCI-H2122, SW1463, SW1573, SW837, and UM-UC-3	Ostrem et al. (2013), Canon et al. (2019)
MRTX849		H1792, H358, H23, Calu-1, MIA-PaCa2, H1373, H2122, SW1573, H2030, and KYSE-410	Ostrem et al. (2013), Hallin et al. (2020)
Modified farnesyltransferase inhibitors (FTIs)	Blocks the addition of a prenyl group to prevent Ras–membrane association	PSN-1 and SW-620	Novotny et al. (2017)

ASM, acid sphingomyelinase; NSM, neutral sphingomyelinase; FTIs, farnesyltransferase inhibitors; PtdSer, phosphatidylserine; PM, plasma membrane.

However, PDE6δ interacts with other prenylated small GTPases including H-Ras, N-Ras, and Rap1 (Chandra et al., 2012; Dumbacher et al., 2018), suggesting that the effect of deltarasin may not be K-Ras–specific. Moreover, K-Ras knockout mice have embryonic lethality, whereas PDE6δ knockout mice develop normally (Johnson et al., 1997; Zhang et al., 2007), indicating that K-Ras is active in the absence of PDE6δ. In sum, PDE6δ interaction with K-Ras is a tractable target to inhibit oncogenic K-Ras activity, and further validation on the K-Ras specificity of PDE6δ would promote translation into the clinic.

## Reducing Phosphatidylserine Content at the Inner PM Leaflet Removes K-Ras From the PM

Phosphatidylserine (PtdSer) is an anionic phospholipid synthesized from phosphatidylcholine (PtdCho) and phosphatidylethanolamine (PtdEth) by PtdSer synthase 1 and 2, respectively, in mammalian cells. While PtdSer is found in the ER and mitochondria, it is concentrated in the inner PM *via* mechanisms that are not fully elucidated (Leventis and Grinstein, 2010; Kay and Fairn, 2019). PM PtdSer plays key roles in physiological processes including the clearance of apoptotic cells, coagulation cascade, and recruitment and activation of signaling proteins (Leventis and Grinstein, 2010; Kay and Fairn, 2019). The anionic head group provides a negative electrostatic potential to the inner PM leaflet, which allows interaction with a stretch of positively charged amino acid residues, called PBD, of PM-localized proteins (Yeung et al., 2008). K-Ras binds PtdSer at the inner PM leaflet through the C-terminal PBD concomitantly with the farnesyl moiety, which provides specificity for PtdSer over other anionic phospholipids (Zhou et al., 2017). Recent studies have reported a number of mechanisms that can reduce PM PtdSer content, which in turn inhibits K-Ras–PM localization and oncogenic K-Ras signaling output.

### Phosphatidylinositol 4-Phosphate Regulates the PM Distribution of PtdSer and K-Ras

**FIGURE 2:**
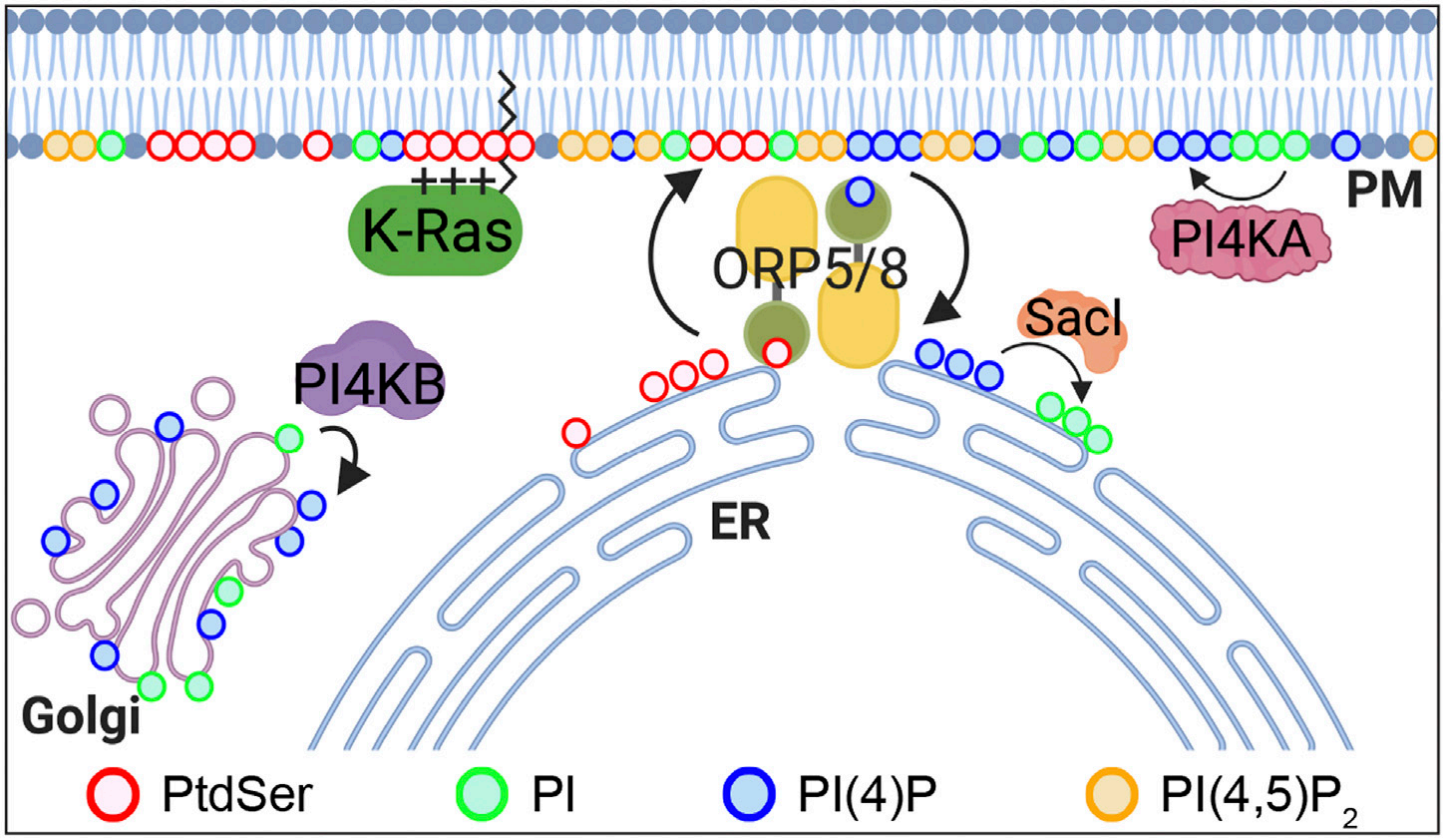
PtdSer PM enrichment is regulated by ORP5 and 8. ORP5 and 8 are lipid transporters that exchange ER PtdSer with PM PI4P. The driving force of this process is a PI4P concentration gradient, whereby PI4P levels are high in the PM by PI4KA and are kept low at the ER by Sac1 phosphatase, which converts PI4P to PI. PI4P is also generated at the Golgi complex by PI4KB. ORP, oxysterol-binding protein–related protein; PtdSer, phosphatidylserine; PI, phosphatidylinositol; PI4P, PI 4-phosphate; PI(4,5)P2, PI(4,5)-bisphosphate; PI4KA, PI 4-kinase IIIα; PI4KB, PI 4-kinase IIIβ.

Phosphatidylinositol (PI) is phosphorylated to PI 4-phosphate (PI4P) by four PI 4-kinases in mammalian cells: PI4K IIα and β (PI4K2A and 2B) and PI4K IIIα and β (PI4KA and PI4KB) (Balla, 2013). PI4KA and 2B localize primarily to the PM, whereas PI4K2A and PI4KB localize to the Golgi complex (Balla, 2013). In mammalian cells, oxysterol-binding protein–related proteins (ORPs) 5 and 8 exchange newly synthesized PtdSer from the ER for PI4P from the PM at ER–PM membrane contacting sites (MCSs) (Figure 2) (Chung et al., 2015; Moser von Filseck et al., 2015). This process is maintained by PM PI4P by PI4KA and the concomitant PI4P hydrolysis by Sac1 phosphatase in the ER to keep a PI4P concentration gradient across the PM and ER (Chung et al., 2015; Moser von Filseck et al., 2015). ORP5 and 8 recruitment to ER–PM MCSs further requires additional PM PI(4,5)P_2_ (Ghai et al., 2017; Sohn et al., 2018). Several studies have reported that perturbing this exchange process reduces PM PtdSer content and inhibits K-Ras–PM binding and K-Ras signal output. PI(4,5)P_2_ reduction by the rapamycin-recruitable 5-phosphatase domain of INPP5E to the PM blocks ORP5 and 8 recruitment to ER–PM MCSs, whereas increasing the PM PI(4,5)P_2_ level by overexpressing PI4P 5-kinase (PIP5K) β reduces PM PI4P levels. In both cases, the exchange of ER PtdSer for PM PI4P is perturbed, resulting in PtdSer reduction in the inner PM leaflet (Ghai et al., 2017; Sohn et al., 2018). Also, the acute depletion of PM PI4P by rapamycin-recruitable Sac1 dissociates K-Ras, but not H-Ras, from the PM and inhibits K-Ras signaling (Gulyas et al., 2017). Ras proteins are spatially organized into nanoscale domains on the PM, called nanoclusters, which are critical for high-fidelity Ras signal output (Prior et al., 2003; Tian et al., 2007; Cho et al., 2012a; Cho and Hancock, 2013). PM PI4P depletion by either ORP5 or 8 knockdown or chemical inhibition redistributes PtdSer and K-Ras from the PM. It further disrupts K-Ras nanoclustering and abrogates K-Ras signal output and the growth of K-Ras–driven pancreatic cancer cells (Kattan et al., 2019). Consistently, ORP5 and 8 are highly expressed in certain types of cancer and involved in the prognosis of cancer patients. A high expression of ORP8 is observed in lung cancer tissues and hamster bile duct cancers in comparison to normal tissues (Fournier et al., 1999; Loilome et al., 2006). ORP5 overexpression enhances the invasion of pancreatic cancer cells, while ORP5 knockdown abrogates it *in vitro*. Moreover, the ORP5 mRNA level is significantly elevated in tumors harboring oncogenic mutant K-Ras compared with tumors with wild-type (WT) K-Ras in cohorts of pancreatic cancer, NSCLC, and 33 types of cancer in the TCGA (the Cancer Genome Atlas) database (Kattan et al., 2019). Further analysis of overall survival periods for patients in these three cohorts demonstrates that cancer patients with low ORP5 or 8 expression have better prognosis than patients with high ORP5 or 8 expression (Koga et al., 2008; Kattan et al., 2019).

In addition to PM PI4P, a recent study has demonstrated that Golgi PI4P is involved in the PM localization of PtdSer and K-Ras. Chemical inhibition of PI4KB, which depletes PI4P at the Golgi complex, but not the PM, translocates K-Ras and PtdSer from the PM to the mitochondria and endomembrane, respectively (Miller et al., 2019). Supplementation with exogenous PtdSer acutely returns K-Ras to the PM in Golgi PI4P–depleted cells, and mitochondrial PtdSer reduction by overexpressing PtdSer decarboxylase, which converts PtdSer to PtdEth at the mitochondria (Percy et al., 1983), redistributes K-Ras from the mitochondria to the endomembranes in Golgi PI4P–depleted cells (Miller et al., 2019). Furthermore, Golgi PI4P depletion inhibits Ras signaling in K-Ras–transformed but not H-Ras–transformed cells. Although the exact mechanism is yet to be elucidated, these data suggest that Golgi PI4P regulates the PM enrichment of PtdSer and thereby K-Ras–PM localization and K-Ras signaling (Miller et al., 2019). In sum, the PtdSer/PI4P exchange mechanism at the ER–PM MCSs, which regulates the PM enrichment of PtdSer and thereby K-Ras–PM localization and signaling, is a viable target for developing anti–K-Ras therapies.

### Perturbing Recycling Endosomal Activity Mislocalizes PtdSer and K-Ras From the PM

In addition to the non-vesicular transport of PtdSer by ORP5 and 8, PtdSer transports *via* the classical vesicular trafficking. Once PM PtdSer is endocytosed, it enters the sorting endosomes, where it either returns to the PM *via* the RE or is transported to lysosomes for its degradation by phospholipases (Leventis and Grinstein, 2010), suggesting that recycling endosomal activity is important for maintaining PM PtdSer content. Recent studies have reported that disruption of recycling endosomal activity depletes PtdSer and K-Ras from the PM. Acylpeptide hydrolase (APEH) removes the N-terminal acylated amino acids from acetylated proteins, and regulates the ubiquitin-mediated protein degradation (Shimizu et al., 2004). APEH knockdown or inhibition blocks endocytic recycling of the transferrin receptor (TfR) and EGFR and mislocalizes K-Ras and PtdSer from the PM (Tan et al., 2019). It also reduces nanoclustering of oncogenic K-Ras that remained at the PM and prevents oncogenic K-Ras signaling and growth of pancreatic cancer cells harboring oncogenic mutant K-Ras but not WT K-Ras. This study proposes that failure to maintain PtdSer and K-Ras at the PM in APEH-depleted cells is in part induced by aberrant RE function.

A protein kinase C (PKC) inhibitor, staurosporine, and its analogs accumulate PtdSer internalized from the PM in the RE, resulting in PM PtdSer depletion in a PKC-independent manner (Cho et al., 2012b). These compounds also induce K-Ras–PM dissociation and disrupt K-Ras PM nanoclustering (Cho et al., 2012b). Consistent with this, they abrogate K-Ras signaling and cell proliferation in K-Ras–transformed cells. Taken all together, perturbing recycling endosomal activity could prevent PM PtdSer replenishment through the RE, which results in K-Ras–PM dissociation and disrupted K-Ras nanoclustering and K-Ras signaling. The perturbed recycling endosomal activity could also block the PDE6δ/RE-mediated K-Ras–PM localization, further contributing to disrupted K-Ras–PM localization and signaling.

### K-Ras and PtdSer PM Localization Is Regulated by Sphingomyelin/Ceramide Biosynthesis

Recent studies have demonstrated that perturbing the enzymes involved in sphingomyelin (SM) metabolism depletes the PM localization of PtdSer and K-Ras, and blocks oncogenic K-Ras signaling. Ceramide, which is synthesized in the ER, trafficks to the Golgi complex, where it is converted to SM. SM is further transported to the PM and lysosomes, where it is reverted to ceramide by sphingomyelinases (Gault et al., 2010). Several studies have reported that the inhibition of acid or neutral sphingomyelinase (ASM and NSM, respectively) dissociates PtdSer and K-Ras from the PM and inhibits oncogenic K-Ras signal transduction (Figure 1). A wide range of ASM inhibitors including tricyclic antidepressants elevates cellular SM contents and accumulates SM in vesicular structures. They also deplete PM PtdSer content and translocate K-Ras, but not H-Ras, from the PM to endomembranes (van der Hoeven et al., 2013; Cho et al., 2016; van der Hoeven et al., 2018). Also, K-Ras is dissociated from the PM in patient-derived Niemann–Pick type A and B cell lines, in which *SMPD1* gene–encoding ASM has inactivating and partial loss-of-function mutations, respectively (Cho et al., 2016; Schuchman and Desnick, 2017). These inhibitors further perturb oncogenic K-Ras PM nanoclustering and its signaling, and abrogate the growth of different types of human cancer cells expressing oncogenic mutant K-Ras but not WT K-Ras (Petersen et al., 2013; van der Hoeven et al., 2013; van der Hoeven et al., 2018). Supplementing ASM-inhibited cells with recombinant ASM returns PtdSer and K-Ras to the PM. Also, replenishing PM PtdSer content with exogenous PtdSer supplementation returns K-Ras to the PM and restores nanoclustering in ASM-inhibited cells, which indicates that K-Ras–PM dissociation occurs through PM PtdSer depletion (Cho et al., 2016). In addition, pharmacological inhibitors for enzymes in the SM/ceramide metabolic pathway redistribute PtdSer and K-Ras from the PM (van der Hoeven et al., 2018). They further perturb K-Ras nanoclustering and block the growth of pancreatic cancer cells harboring oncogenic mutant K-Ras (van der Hoeven et al., 2018). In a supplemental *C. elegans* study, RNAi-mediated knockdown of 14 genes encoding enzymes in the SM/ceramide biosynthesis pathway suppressed the LET-60G13D (a K-RasG13D ortholog in *C. elegans*)-induced multi-vulva phenotype (van der Hoeven et al., 2018).

Another approach to disrupt SM/ceramide metabolism is to alter the activity of NSM. Avicins, natural plant-derived triterpenoid saponins from *Acacia victoriae*, have proapoptotic, anti-inflammatory, and anticancer activities (Wang et al., 2010). A recent study demonstrated that avicin G, an isomer of avicin compounds, inhibits NSM and ASM, with a greater potency against NSM, and elevates cellular SM, ceramide, and PtdSer contents (Garrido et al., 2020). It also disrupts endosomal recycling of the EGFR and perturbs lysosomal activity by elevating the lysosomal pH (Garrido et al., 2020). Avicin G and other NSM inhibitors redistribute PtdSer from the PM, accumulate K-Ras in lysosomes, and increase the K-Ras protein level. Since K-Ras and PtdSer are proposed to be degraded in the lysosome (Lu et al., 2009; Leventis and Grinstein, 2010), the elevated K-Ras and PtdSer levels induced by avicin G, in part, account for the perturbed lysosomal activity (Garrido et al., 2020). It further perturbs K-Ras PM nanoclustering and blocks K-Ras signaling and the growth of K-Ras–addicted pancreatic and NSCLC cell lines (Garrido et al., 2020). Taken together, these studies propose that a correct SM/ceramide balance maintains the PM localization of PtdSer and K-Ras and that pharmacological agents that perturb the sphingolipid pathways could be a new strategy for developing anti–K-Ras therapies (van der Hoeven et al., 2018). One plausible mechanism of PM PtdSer depletion by altering the cellular SM contents is through perturbing recycling endosomal activity. The RE is enriched with cholesterol, SM, and PtdSer (Gagescu et al., 2000; Uchida et al., 2011), and elevating cellular sphingolipid contents blocks endosomal recycling of the glucose transporter 1 and TfR (Finicle et al., 2018). Like avicin G, staurosporine and its analogs perturb the RE activity and elevate cellular SM content in a PKC-independent manner by reducing the protein level of ORMDL, which negatively regulates serine-palmitoyltransferase, the rate-limiting enzyme for sphingolipid biosynthesis (Maekawa et al., 2016). Taken all together, it is proposed that an increased cellular SM level changes SM content at the RE, which disrupts recycling endosomal activity. This, in turn, depletes PtdSer and mislocalizes K-Ras from the PM, as discussed above.

## Conclusion

Despite the essential role of oncogenic mutant K-Ras in the growth and survival of pancreatic, lung, and colorectal cancers, there are no anti–K-Ras therapies available in the clinic. Several studies have reported that knockdown of endogenous oncogenic mutant K-Ras in a range of NCSLC and pancreatic cancer cell lines blocks their growth and survival, suggesting that blocking oncogenic K-Ras activity is a valid strategy for anti–K-Ras therapies. Ras drug discovery efforts have focused largely on inhibitors of Ras downstream effectors including B-Raf, C-Raf, PI3K, and MEK (Baines et al., 2011). One example is the multikinase inhibitor, Nexavar, used against renal cell and hepatocellular carcinoma (Llovet et al., 2008; Roberts, 2008), although it is unclear to what extent the efficacy of Nexavar towards these cancers is related to the inhibition of C-Raf, B-Raf, or VEGFR (Downward, 2003; Baines et al., 2011). B-Raf–specific inhibitors produce excellent, albeit often short-lived, responses in patients with B-Raf mutant melanoma (Flaherty et al., 2010). However, further studies have shown that B-Raf–specific inhibitors paradoxically activate the MAPK cascade in melanoma cells expressing oncogenic mutant N- or K-Ras *via* a mechanism that involves C-Raf hyperactivation (Heidorn et al., 2010; Cho et al., 2012a). These studies illustrate that blocking MAPK signaling with Raf kinase inhibitors is a limited approach to anti-Ras therapy.

Recently, two small molecules that directly bind and inhibit the K-RasG12C mutant have shown promising outcomes in clinical trials. While the K-RasG12C mutant is found in a small fraction of K-Ras–driven human cancers, these studies demonstrate that developing anti–K-Ras therapies is feasible. One approach to inhibit all oncogenic mutant K-Ras is to block its interaction with the PM since K-Ras must localize to the PM for its signal transduction. However, the exact molecular mechanisms of K-Ras transport to and maintenance at the PM are not fully elucidated. In this review, we discussed several recently identified mechanisms that regulate K-Ras–PM interaction and thereby the K-Ras signal cascade. Compounds that perturb these mechanisms dissociate K-Ras from the PM and block K-Ras signaling and K-Ras–dependent cancer cell growth. However, this approach has pitfalls including nonspecificity and cytotoxicity since it does not specifically target K-Ras. For example, PDE6δ can bind other farnesylated small GTPases *via* the same hydrophobic pocket as K-Ras. Thus, blocking this binding site by PDE6δ inhibitors can dysregulate the cellular localizations and activities of K-Ras and other small GTPases. Also, PtdSer at the inner PM leaflet recruits and promotes the activity of K-Ras and other proteins containing a polybasic domain (Leventis and Grinstein, 2010; Kay and Fairn, 2019). While PM PI4P regulates the PM enrichments of PtdSer, it can be further phosphorylated to different PIPs, which activate several essential signaling proteins (Balla, 2013). Therefore, while depleting PM PtdSer or perturbing the PI4P/PtdSer exchange mechanism prevents oncogenic mutant K-Ras activity, they can also perturb other essential signaling cascades. Nevertheless, many studies have reported that disrupting these molecular mechanisms blocks the growth of human cancer cells that are K-Ras–dependent but not K-Ras–independent *in vitro* and *in vivo*, suggesting that targeting these mechanisms is a valid approach for developing anti–K-Ras therapies.

Cancer chemotherapy is most effective when a combination of drugs targeting different molecular mechanisms are applied. There are four major approaches that are currently being perused for developing anti–K-Ras therapies, and any one approach alone may not be sufficient to completely block oncogenic K-Ras signaling due to high cytotoxicity and/or nonspecificity. A recent study has demonstrated that a K-RasG12C inhibitor potentiates the anticancer effect of the MEK, mTOR, and insulin-like growth factor 1 receptor (IFG1R) inhibitors in NSCLC cells. While combined mTOR, IGF1R, and MEK inhibition shows significant tumor regression in K-RasG12C–driven lung cancer mouse models, replacing the MEK inhibitor with a K-RasG12C inhibitor in combination demonstrates greater efficacy, specificity, and tolerability (Molina-Arcas et al., 2019). Moreover, the combination of the K-RasG12C inhibitor with anti–PD-1 immune checkpoint inhibition synergistically suppresses tumor growth in K-RasG12C–driven mouse models (Canon et al., 2019). Combination therapy of K-RasG12C inhibitors with anti–PD-1 or anti–PD-L1 in patients with solid tumors harboring the K-RasG12C mutant is currently in clinical trials (ClinicalTrials.gov identifier: NCT04185883, NCT03785249). Although combination therapy with K-RasG12C inhibitors and other anticancer approaches is promising, it is limited to K-RasG12C–specific cancers, which accounts for ∼20% of K-Ras–driven cancers. Therefore, it would be worthwhile to examine the effects of combining pharmacological agents that can block all oncogenic mutant K-Ras by dissociating it from the PM with drugs developed for targeting the other approaches.

## References

[B1] Apolloni A. Prior I. A. Lindsay M. Parton R. G. Hancock J. F. (2000). H-ras but Not K-Ras Traffics to the Plasma Membrane through the Exocytic Pathway. Mol. Cel. Biol. 20 (7), 2475–2487. 10.1128/mcb.20.7.2475-2487.2000 PMC85443 10713171

[B2] Baines A. T. Xu D. Der C. J. (2011). Inhibition of Ras for Cancer Treatment: the Search Continues. Future Med. Chem. 3 (14), 1787–1808. 10.4155/fmc.11.121 22004085 PMC3347641

[B3] Balla T. (2013). Phosphoinositides: Tiny Lipids with Giant Impact on Cell Regulation. Physiol. Rev. 93 (3), 1019–1137. 10.1152/physrev.00028.2012 23899561 PMC3962547

[B4] Barbacid M. (1987). Ras Genes. Annu. Rev. Biochem. 56, 779–827. 10.1146/annurev.bi.56.070187.004023 3304147

[B5] Canon J. Rex K. Saiki A. Y. Mohr C. Cooke K. Bagal D. (2019). The Clinical KRAS(G12C) Inhibitor AMG 510 Drives Anti-tumour Immunity. Nature. 575 (7781), 217–223. 10.1038/s41586-019-1694-1 31666701

[B6] Chandra A. Grecco H. E. Pisupati V. Perera D. Cassidy L. Skoulidis F. (2012). The GDI-like Solubilizing Factor PDEδ Sustains the Spatial Organization and Signalling of Ras Family Proteins. Nat. Cel Biol. 14 (2), 148–158. 10.1038/ncb2394 22179043

[B7] Cho K. J. Hancock J. F. (2013). Ras Nanoclusters: a New Drug Target? Small GTPases. 4 (1), 57–60. 10.4161/sgtp.23145 23419283 PMC3620104

[B8] Cho K. J. Kasai R. S. Park J. H. Chigurupati S. Heidorn S. J. van der Hoeven D. (2012). Raf Inhibitors Target Ras Spatiotemporal Dynamics. Curr. Biol. 22 (11), 945–955. 10.1016/j.cub.2012.03.067 22560614

[B9] Cho K. J. Park J. H. Piggott A. M. Salim A. A. Gorfe A. A. Parton R. G. (2012). Staurosporines Disrupt Phosphatidylserine Trafficking and Mislocalize Ras Proteins. J. Biol. Chem. 287 (52), 43573–43584. 10.1074/jbc.M112.424457 23124205 PMC3527944

[B10] Cho K. J. van der Hoeven D. Zhou Y. Maekawa M. Ma X. Chen W. (2016). Inhibition of Acid Sphingomyelinase Depletes Cellular Phosphatidylserine and Mislocalizes K-Ras from the Plasma Membrane. Mol. Cel Biol. 36 (2), 363–374. 10.1128/MCB.00719-15 PMC4719297 26572827

[B11] Chung J. Torta F. Masai K. Lucast L. Czapla H. Tanner L. B. (2015). INTRACELLULAR TRANSPORT. PI4P/phosphatidylserine Countertransport at ORP5- and ORP8-Mediated ER-Plasma Membrane Contacts. Science. 349 (6246), 428–432. 10.1126/science.aab1370 26206935 PMC4638224

[B12] Cohen S. J. Ho L. Ranganathan S. Abbruzzese J. L. Alpaugh R. K. Beard M. (2003). Phase II and Pharmacodynamic Study of the Farnesyltransferase Inhibitor R115777 as Initial Therapy in Patients with Metastatic Pancreatic Adenocarcinoma. J. Clin. Oncol. 21 (7), 1301–1306. 10.1200/JCO.2003.08.040 12663718

[B13] Cox A. D. Fesik S. W. Kimmelman A. C. Luo J. Der C. J. (2014). Drugging the Undruggable RAS: Mission Possible? Nat. Rev. Drug Discov. 13 (11), 828–851. 10.1038/nrd4389 25323927 PMC4355017

[B14] Downward J. (2003). Targeting RAS Signalling Pathways in Cancer Therapy. Nat. Rev. Cancer 3 (1), 11–22. 10.1038/nrc969 12509763

[B15] Dumbacher M. Van Dooren T. Princen K. De Witte K. Farinelli M. Lievens S. (2018). Modifying Rap1-Signalling by Targeting Pde6delta Is Neuroprotective in Models of Alzheimer's Disease. Mol. Neurodegener. 13 (1), 50. 10.1186/s13024-018-0283-3 30257685 PMC6158915

[B16] Esteban L. M. Vicario-Abejón C. Fernández-Salguero P. Fernández-Medarde A. Swaminathan N. Yienger K. (2001). Targeted Genomic Disruption of H-Ras and N-Ras, Individually or in Combination, Reveals the Dispensability of Both Loci for Mouse Growth and Development. Mol. Cel. Biol. 21 (5), 1444–1452. 10.1128/mcb.21.5.1444-1452.2001 PMC86690 11238881

[B17] Finicle B. T. Ramirez M. U. Liu G. Selwan E. M. McCracken A. N. Yu J. (2018). Sphingolipids Inhibit Endosomal Recycling of Nutrient Transporters by Inactivating ARF6. J. Cel Sci. 131 (12). 10.1242/jcs.213314 PMC6031383 29848659

[B18] Flaherty K. T. Puzanov I. Kim K. B. Ribas A. McArthur G. A. Sosman J. A. (2010). Inhibition of Mutated, Activated BRAF in Metastatic Melanoma. N. Engl. J. Med. 363 (9), 809–819. 10.1056/NEJMoa1002011 20818844 PMC3724529

[B19] Fournier M. V. Guimaraes da Costa F. Paschoal M. E. Ronco L. V. Carvalho M. G. Pardee A. B. (1999). Identification of a Gene Encoding a Human Oxysterol-Binding Protein-Homologue: a Potential General Molecular Marker for Blood Dissemination of Solid Tumors. Cancer Res. 59 (15), 3748–3753. 10446991

[B20] Gagescu R. Demaurex N. Parton R. G. Hunziker W. Huber L. A. Gruenberg J. (2000). The Recycling Endosome of Madin-Darby Canine Kidney Cells Is a Mildly Acidic Compartment Rich in Raft Components. Mol. Biol. Cel. 11 (8), 2775–2791. 10.1091/mbc.11.8.2775 PMC14955 10930469

[B21] Garrido C. M. Henkels K. M. Rehl K. M. Liang H. Zhou Y. Gutterman J. U. (2020). Avicin G Is a Potent Sphingomyelinase Inhibitor and Blocks Oncogenic K- and H-Ras Signaling. Sci. Rep. 10 (1), 9120. 10.1038/s41598-020-65882-5 32499517 PMC7272413

[B22] Gault C. R. Obeid L. M. Hannun Y. A. (2010). An Overview of Sphingolipid Metabolism: from Synthesis to Breakdown. Adv. Exp. Med. Biol. 688, 1–23. 10.1007/978-1-4419-6741-1_1 20919643 PMC3069696

[B23] Ghai R. Du X. Wang H. Dong J. Ferguson C. Brown A. J. (2017). ORP5 and ORP8 Bind Phosphatidylinositol-4, 5-biphosphate (PtdIns(4,5)P 2) and Regulate its Level at the Plasma Membrane. Nat. Commun. 8 (1), 757. 10.1038/s41467-017-00861-5 28970484 PMC5624964

[B24] Gulyas G. Radvanszki G. Matuska R. Balla A. Hunyady L. Balla T. (2017). Plasma Membrane Phosphatidylinositol 4-phosphate and 4,5-bisphosphate Determine the Distribution and Function of K-Ras4B but Not H-Ras Proteins. J. Biol. Chem. 292 (46), 18862–18877. 10.1074/jbc.M117.806679 28939768 PMC5704471

[B25] Hallin J. Engstrom L. D. Hargis L. Calinisan A. Aranda R. Briere D. M. (2020). The KRAS(G12C) Inhibitor MRTX849 Provides Insight toward Therapeutic Susceptibility of KRAS-Mutant Cancers in Mouse Models and Patients. Cancer Discov. 10 (1), 54–71. 10.1158/2159-8290.CD-19-1167 31658955 PMC6954325

[B26] Hancock J. F. Magee A. I. Childs J. E. Marshall C. J. (1989). All Ras Proteins Are Polyisoprenylated but Only Some Are Palmitoylated. Cell. 57 (7), 1167–1177. 10.1016/0092-8674(89)90054-8 2661017

[B27] Hancock J. F. Paterson H. Marshall C. J. (1990). A Polybasic Domain or Palmitoylation Is Required in Addition to the CAAX Motif to Localize P21ras to the Plasma Membrane. Cell. 63 (1), 133–139. 10.1016/0092-8674(90)90294-o 2208277

[B28] Hancock J. F. (2003). Ras Proteins: Different Signals from Different Locations. Nat. Rev. Mol. Cel Biol. 4 (5), 373–385. 10.1038/nrm1105 12728271

[B29] Hayes T. K. Neel N. F. Hu C. Gautam P. Chenard M. Long B. (2016). Long-Term ERK Inhibition in KRAS-Mutant Pancreatic Cancer Is Associated with MYC Degradation and Senescence-like Growth Suppression. Cancer Cell. 29 (1), 75–89. 10.1016/j.ccell.2015.11.011 26725216 PMC4816652

[B30] Heidorn S. J. Milagre C. Whittaker S. Nourry A. Niculescu-Duvas I. Dhomen N. (2010). Kinase-dead BRAF and Oncogenic RAS Cooperate to Drive Tumor Progression through CRAF. Cell. 140 (2), 209–221. 10.1016/j.cell.2009.12.040 20141835 PMC2872605

[B31] Ismail S. A. Chen Y.-X. Rusinova A. Chandra A. Bierbaum M. Gremer L. (2011). Arl2-GTP and Arl3-GTP Regulate a GDI-like Transport System for Farnesylated Cargo. Nat. Chem. Biol. 7 (12), 942–949. 10.1038/nchembio.686 22002721

[B32] Johnson L. Greenbaum D. Cichowski K. Mercer K. Murphy E. Schmitt E. (1997). K-ras Is an Essential Gene in the Mouse with Partial Functional Overlap with N-Ras. Genes Development. 11 (19), 2468–2481. 10.1101/gad.11.19.2468 9334313 PMC316567

[B33] Kattan W. E. Chen W. Ma X. Lan T. H. van der Hoeven D. van der Hoeven R. (2019). Targeting Plasma Membrane Phosphatidylserine Content to Inhibit Oncogenic KRAS Function. Life Sci. Alliance. 2 (5), e201900431. 10.26508/lsa.201900431 31451509 PMC6709719

[B34] Kay J. G. Fairn G. D. (2019). Distribution, Dynamics and Functional Roles of Phosphatidylserine within the Cell. Cell Commun Signal. 17 (1), 126. 10.1186/s12964-019-0438-z 31615534 PMC6792266

[B35] Kim E. Ambroziak P. Otto J. C. Taylor B. Ashby M. Shannon K. (1999). Disruption of the Mouse Rce1 Gene Results in Defective Ras Processing and Mislocalization of Ras within Cells. J. Biol. Chem. 274 (13), 8383–8390. 10.1074/jbc.274.13.8383 10085069

[B36] Klein C. H. Truxius D. C. Vogel H. A. Harizanova J. Murarka S. Martin-Gago P. (2019). PDEdelta Inhibition Impedes the Proliferation and Survival of Human Colorectal Cancer Cell Lines Harboring Oncogenic KRas. Int. J. Cancer. 144 (4), 767–776. 10.1002/ijc.31859 30194764 PMC6519276

[B37] Koera K. Nakamura K. Nakao K. Miyoshi J. Toyoshima K. Hatta T. (1997). K-ras Is Essential for the Development of the Mouse Embryo. Oncogene. 15 (10), 1151–1159. 10.1038/sj.onc.1201284 9294608

[B38] Koga Y. Ishikawa S. Nakamura T. Masuda T. Nagai Y. Takamori H. (2008). Oxysterol Binding Protein-Related Protein-5 Is Related to Invasion and Poor Prognosis in Pancreatic Cancer. Cancer Sci. 99 (12), 2387–2394. 10.1111/j.1349-7006.2008.00987.x 19032366 PMC11159934

[B39] Lau H. Y. Ramanujulu P. M. Guo D. Yang T. Wirawan M. Casey P. J. (2014). An Improved Isoprenylcysteine Carboxylmethyltransferase Inhibitor Induces Cancer Cell Death and Attenuates Tumor Growth *In Vivo* . Cancer Biol. Ther. 15 (9), 1280–1291. 10.4161/cbt.29692 24971579 PMC4128870

[B40] Leung E. L. H. Luo L. X. Liu Z. Q. Wong V. K. W. Lu L. L. Xie Y. (2018). Inhibition of KRAS-dependent Lung Cancer Cell Growth by Deltarasin: Blockage of Autophagy Increases its Cytotoxicity. Cell Death Dis. 9 (2), 216. 10.1038/s41419-017-0065-9 29440631 PMC5833846

[B41] Leventis P. A. Grinstein S. (2010). The Distribution and Function of Phosphatidylserine in Cellular Membranes. Annu. Rev. Biophys. 39, 407–427. 10.1146/annurev.biophys.093008.131234 20192774

[B42] Llovet J. M. Ricci S. Mazzaferro V. Hilgard P. Gane E. Blanc J. F. (2008). Sorafenib in Advanced Hepatocellular Carcinoma. N. Engl. J. Med. 359 (4), 378–390. 10.1056/NEJMoa0708857 18650514

[B43] Loilome W. Yongvanit P. Wongkham C. Tepsiri N. Sripa B. Sithithaworn P. (2006). Altered Gene Expression in Opisthorchis Viverrini-Associated Cholangiocarcinoma in Hamster Model. Mol. Carcinog. 45 (5), 279–287. 10.1002/mc.20094 16550611

[B44] Lu A. Tebar F. Alvarez-Moya B. Lopez-Alcala C. Calvo M. Enrich C. (2009). A clathrin-dependent pathway leads to KRas signaling on late endosomes en route to lysosomes. J. Cel Biol. 184 (6), 863–879. 10.1083/jcb.200807186 PMC2699148 19289794

[B45] Macdonald J. S. McCoy S. Whitehead R. P. Iqbal S. Wade J. L. 3rd Giguere J. K. (2005). A Phase II Study of Farnesyl Transferase Inhibitor R115777 in Pancreatic Cancer: a Southwest Oncology Group (SWOG 9924) Study. Invest. New Drugs. 23 (5), 485–487. 10.1007/s10637-005-2908-y 16133800

[B46] Maekawa M. Lee M. Wei K. Ridgway N. D. Fairn G. D. (2016). Staurosporines Decrease ORMDL Proteins and Enhance Sphingomyelin Synthesis Resulting in Depletion of Plasmalemmal Phosphatidylserine. Sci. Rep. 6, 35762. 10.1038/srep35762 27805006 PMC5090970

[B47] Martin-Gago P. Fansa E. K. Klein C. H. Murarka S. Janning P. Schurmann M. (2017). A PDE6delta-KRas Inhibitor Chemotype with up to Seven H-Bonds and Picomolar Affinity that Prevents Efficient Inhibitor Release by Arl2. Angew. Chem. Int. Ed. Engl. 56 (9), 2423–2428. 10.1002/anie.201610957 28106325

[B48] Miller T. E. Henkels K. M. Huddleston M. Salisbury R. Hussain S. M. Sasaki A. T. (2019). Depletion of Phosphatidylinositol 4-phosphate at the Golgi Translocates K-Ras to Mitochondria. J. Cel Sci. 132 (16). 10.1242/jcs.231886 PMC6737909 31331963

[B49] Molina-Arcas M. Moore C. Rana S. van Maldegem F. Mugarza E. Romero-Clavijo P. (2019). Development of Combination Therapies to Maximize the Impact of KRAS-G12c Inhibitors in Lung Cancer. Sci. Transl Med. 11 (510). 10.1126/scitranslmed.aaw7999 PMC6764843 31534020

[B50] Moser von Filseck J. Copic A. Delfosse V. Vanni S. Jackson C. L. Bourguet W. (2015). INTRACELLULAR TRANSPORT. Phosphatidylserine Transport by ORP/Osh Proteins Is Driven by Phosphatidylinositol 4-phosphate. Science. 349 (6246), 432–436. 10.1126/science.aab1346 26206936

[B51] Novotny C. J. Hamilton G. L. McCormick F. Shokat K. M. (2017). Farnesyltransferase-Mediated Delivery of a Covalent Inhibitor Overcomes Alternative Prenylation to Mislocalize K-Ras. ACS Chem. Biol. 12 (7), 1956–1962. 10.1021/acschembio.7b00374 28530791 PMC6070134

[B52] O'Bryan J. P. (2019). Pharmacological Targeting of RAS: Recent success with Direct Inhibitors. Pharmacol. Res. 139, 503–511. 10.1016/j.phrs.2018.10.021 30366101 PMC6360110

[B53] Ostrem J. M. Peters U. Sos M. L. Wells J. A. Shokat K. M. (2013). K-Ras(G12C) Inhibitors Allosterically Control GTP Affinity and Effector Interactions. Nature. 503 (7477), 548–551. 10.1038/nature12796 24256730 PMC4274051

[B54] Papke B. Murarka S. Vogel H. A. Martin-Gago P. Kovacevic M. Truxius D. C. (2016). Identification of Pyrazolopyridazinones as PDEdelta Inhibitors. Nat. Commun. 7, 11360. 10.1038/ncomms11360 27094677 PMC4843002

[B55] Pells S. Divjak M. Romanowski P. Impey H. Hawkins N. J. Clarke A. R. (1997). Developmentally-regulated Expression of Murine K-Ras Isoforms. Oncogene. 15 (15), 1781–1786. 10.1038/sj.onc.1201354 9362444

[B56] Percy A. K. Moore J. F. Carson M. A. Waechter C. J. (1983). Characterization of Brain Phosphatidylserine Decarboxylase: Localization in the Mitochondrial Inner Membrane. Arch. Biochem. Biophys. 223 (2), 484–494. 10.1016/0003-9861(83)90613-6 6859873

[B57] Petersen N. H. Olsen O. D. Groth-Pedersen L. Ellegaard A. M. Bilgin M. Redmer S. (2013). Transformation-associated Changes in Sphingolipid Metabolism Sensitize Cells to Lysosomal Cell Death Induced by Inhibitors of Acid Sphingomyelinase. Cancer Cell. 24 (3), 379–393. 10.1016/j.ccr.2013.08.003 24029234

[B58] Potenza N. Vecchione C. Notte A. De Rienzo A. Rosica A. Bauer L. (2005). Replacement of K‐Ras with H‐Ras Supports normal Embryonic Development Despite Inducing Cardiovascular Pathology in Adult Mice. EMBO Rep. 6 (5), 432–437. 10.1038/sj.embor.7400397 15864294 PMC1299307

[B59] Prior I. A. Hood F. E. Hartley J. L. (2020). The Frequency of Ras Mutations in Cancer. Cancer Res. 80, 2969–2974. 10.1158/0008-5472.CAN-19-3682 32209560 PMC7367715

[B60] Prior I. A. Muncke C. Parton R. G. Hancock J. F. (2003). Direct Visualization of Ras Proteins in Spatially Distinct Cell Surface Microdomains. J. Cel Biol. 160 (2), 165–170. 10.1083/jcb.200209091 PMC2172642 12527752

[B61] Roberts L. R. (2008). Sorafenib in Liver Cancer-Jjust the Beginning. N. Engl. J. Med. 359 (4), 420–422. 10.1056/NEJMe0802241 18650519

[B62] Rocks O. Gerauer M. Vartak N. Koch S. Huang Z.-P. Pechlivanis M. (2010). The Palmitoylation Machinery Is a Spatially Organizing System for Peripheral Membrane Proteins. Cell. 141 (3), 458–471. 10.1016/j.cell.2010.04.007 20416930

[B63] Rocks O. Peyker A. Kahms M. Verveer P. J. Koerner C. Lumbierres M. (2005). An Acylation Cycle Regulates Localization and Activity of Palmitoylated Ras Isoforms. Science. 307 (5716), 1746–1752. 10.1126/science.1105654 15705808

[B64] Schmick M. Vartak N. Papke B. Kovacevic M. Truxius D. C. Rossmannek L. (2014). KRas Localizes to the Plasma Membrane by Spatial Cycles of Solubilization, Trapping and Vesicular Transport. Cell. 157 (2), 459–471. 10.1016/j.cell.2014.02.051 24725411

[B65] Schuchman E. H. Desnick R. J. (2017). Types A and B Niemann-Pick Disease. Mol. Genet. Metab. 120 (1-2), 27–33. 10.1016/j.ymgme.2016.12.008 28164782 PMC5347465

[B66] Shimizu K. Kiuchi Y. Ando K. Hayakawa M. Kikugawa K. (2004). Coordination of Oxidized Protein Hydrolase and the Proteasome in the Clearance of Cytotoxic Denatured Proteins. Biochem. Biophys. Res. Commun. 324 (1), 140–146. 10.1016/j.bbrc.2004.08.231 15464994

[B67] Singh A. Greninger P. Rhodes D. Koopman L. Violette S. Bardeesy N. (2009). A Gene Expression Signature Associated with "K-Ras Addiction" Reveals Regulators of EMT and Tumor Cell Survival. Cancer Cell. 15 (6), 489–500. 10.1016/j.ccr.2009.03.022 19477428 PMC2743093

[B68] Sohn M. Korzeniowski M. Zewe J. P. Wills R. C. Hammond G. R. V. Humpolickova J. (2018). PI(4,5)P2 Controls Plasma Membrane PI4P and PS Levels *via* ORP5/8 Recruitment to ER-PM Contact Sites. J. Cel Biol. 217 (5), 1797–1813. 10.1083/jcb.201710095 PMC5940310 29472386

[B69] Tan L. Cho K. J. Kattan W. E. Garrido C. M. Zhou Y. Neupane P. (2019). Acylpeptide Hydrolase Is a Novel Regulator of KRAS Plasma Membrane Localization and Function. J. Cel Sci. 132 (15). 10.1242/jcs.232132 PMC6703705 31266814

[B70] Tian T. Harding A. Inder K. Plowman S. Parton R. G. Hancock J. F. (2007). Plasma Membrane Nanoswitches Generate High-Fidelity Ras Signal Transduction. Nat. Cel Biol. 9 (8), 905–914. 10.1038/ncb1615 17618274

[B71] Uchida Y. Hasegawa J. Chinnapen D. Inoue T. Okazaki S. Kato R. (2011). Intracellular Phosphatidylserine Is Essential for Retrograde Membrane Traffic through Endosomes. Proc. Natl. Acad. Sci. U S A. 108 (38), 15846–15851. 10.1073/pnas.1109101108 21911378 PMC3179068

[B72] Van Cutsem E. van de Velde H. Karasek P. Oettle H. Vervenne W. L. Szawlowski A. (2004). Phase III Trial of Gemcitabine Plus Tipifarnib Compared with Gemcitabine Plus Placebo in Advanced Pancreatic Cancer. J. Clin. Oncol. 22 (8), 1430–1438. 10.1200/JCO.2004.10.112 15084616

[B73] van der Hoeven D. Cho K. J. Ma X. Chigurupati S. Parton R. G. Hancock J. F. (2013). Fendiline Inhibits K-Ras Plasma Membrane Localization and Blocks K-Ras Signal Transmission. Mol. Cel Biol. 33 (2), 237–251. 10.1128/MCB.00884-12 PMC3554123 23129805

[B74] van der Hoeven D. Cho K. J. Zhou Y. Ma X. Chen W. Naji A. (2018). Sphingomyelin Metabolism Is a Regulator of K-Ras Function. Mol. Cel Biol 38 (3), e00373. 10.1128/MCB.00373-17 PMC5770534 29158292

[B75] Wang H. Haridas V. Gutterman J. U. Xu Z. X. (2010). Natural Triterpenoid Avicins Selectively Induce Tumor Cell Death. Commun. Integr. Biol. 3 (3), 205–208. 10.4161/cib.3.3.11492 20714394 PMC2918757

[B76] Weinstein I. B. Joe A. (2008). Oncogene Addiction. Cancer Res. 68 (9), 3077–3080. 10.1158/0008-5472.CAN-07-3293 18451130

[B77] Willumsen B. M. Christensen A. Hubbert N. L. Papageorge A. G. Lowy D. R. (1984). The P21 Ras C-Terminus Is Required for Transformation and Membrane Association. Nature. 310 (5978), 583–586. 10.1038/310583a0 6087162

[B78] Yeung T. Gilbert G. E. Shi J. Silvius J. Kapus A. Grinstein S. (2008). Membrane Phosphatidylserine Regulates Surface Charge and Protein Localization. Science. 319 (5860), 210–213. 10.1126/science.1152066 18187657

[B79] Zhang H. Li S. Doan T. Rieke F. Detwiler P. B. Frederick J. M. (2007). Deletion of PrBP/delta Impedes Transport of GRK1 and PDE6 Catalytic Subunits to Photoreceptor Outer Segments. Proc. Natl. Acad. Sci. U S A. 104 (21), 8857–8862. 10.1073/pnas.0701681104 17496142 PMC1885592

[B80] Zhang X. Yu L. Xu H. (2016). Lysosome Calcium in ROS Regulation of Autophagy. Autophagy. 12 (10), 1954–1955. 10.1080/15548627.2016.1212787 27485905 PMC5079666

[B81] Zhou Y. Prakash P. Liang H. Cho K.-J. Gorfe A. A. Hancock J. F. (2017). Lipid-Sorting Specificity Encoded in K-Ras Membrane Anchor Regulates Signal Output. Cell. 168 (1-2), 239–251 e16. 10.1016/j.cell.2016.11.059 28041850 PMC5653213

[B82] Zimmermann G. Papke B. Ismail S. Vartak N. Chandra A. Hoffmann M. (2013). Small Molecule Inhibition of the KRAS-PDEdelta Interaction Impairs Oncogenic KRAS Signalling. Nature. 497 (7451), 638–642. 10.1038/nature12205 23698361

